# Early heat shock protein 72 and 90α intracellular and extracellular responses in patients with severe sepsis or systemic inflammatory response syndrome

**DOI:** 10.1186/cc14121

**Published:** 2015-03-16

**Authors:** K Apostolou, K Vardas, E Briassouli, K Psara, D Goukos, E Mageira, S Nanas, C Routsi, G Briassoulis

**Affiliations:** 1 Evangelismos Hospital, National and Kapodistrian University of Athens, Greece; 2 University Hospital, Heraklion, Greece

## Introduction

Heat shock proteins (HSPs) have intracellular cytopro tective actions, while they act extracellularly as inducers of cytokines and stimulants for immune cells during stress. Their induction constitutes a highly conserved cellular defense mechanism against all kinds of stress. Our objective was to determine the intracellular as well as extracellular levels of HSP72 and HSP90α in patients with severe sepsis (SS) or systemic inflammatory response syndrome (SIRS) admitted to a general ICU, compared with those of healthy individuals; to correlate their expression with severity of illness.

## Methods

Eighty-two consecutively admitted patients in the ICU (35 SIRS, 47 SS) as well as 35 healthy controls (H) were finally enrolled in the study. Patients' demographic characteristics, laboratory examinations and Acute Physiology and Chronic Health Evaluation (APACHE II) score were recorded on admission. HSP levels were determined intracellularly using four-color flow cytometry. Mean fluorescence intensity (MFI) values for each HSP were measured and analyzed. Extracellular levels of HSPs were determined via ELISA.

## Results

HSP expression differed significantly between groups (Kruskal-Wallis), both intracellularly (HSP72 lower in SS, *P *< 0.001), and extracellularly (higher levels of HSP90α (*P *< 0.001) and HSP72 (*P *= 0.003) in SS). HSP72 and HSP90α intracellular expression was inversely correlated to severity of illness, as expressed by APACHE II score (Spearman's, *P *= 0.003 and *P *= 0.025 respectively). Intracellular HSP72 was correlated to mortality when confounding factors were excluded from the analysis (logistic regression, *P *= 0.05). Extracellular HSP90α levels correlated with prolonged PT (*P *= 0.021) and INR (*P *= 0.008). Finally, in the SIRS group, intracellular levels of HSP90α were higher in nonsurvivors (*P *< 0.001).

## Conclusion

SS is characterized by high levels of extracellular HSPs. Intracellular HSP72 is highly expressed during the acute phase of stress in SIRS, while being downregulated in SS. HSP72 and HSP90α intracellular expression and extracellular level variations correlate with severity of illness and mortality.

## Acknowledgements

This research was co-financed by the European Union (European Social Fund) and Greek national funds through the Operational Program 'Education and Lifelong Learning' of the National Strategic Reference Framework Research Funding Program: THALES.

